# β-lactam antibiotic-induced release of lipoteichoic acid from *Staphylococcus aureus *leads to activation of neutrophil granulocytes

**DOI:** 10.1186/1476-0711-5-15

**Published:** 2006-06-27

**Authors:** Sonja Lotz, Andrea Starke, Christian Ziemann, Siegfried Morath, Thomas Hartung, Werner Solbach, Tamás Laskay

**Affiliations:** 1 Institute for Medical Microbiology and Hygiene, University of Lübeck, Germany; 2 ECVAM, Joint Research Center, Ispra, Italy; 3 Department of Biochemical Pharmacology, University of Konstanz, Germany

## Abstract

**Background:**

Polymorphonuclear neutrophil granulocytes (PMN) are phagocytes of the first line of antimicrobial defense. Previously we demonstrated that lipoteichoic acid (LTA) from *Staphylococcus aureus *(*S. aureus*) directly activates neutrophil granulocytes. Others have reported that exposure of *S. aureus *to β-lactam antibiotics leads to LTA release. In the present study we addressed the question whether exposure of *S. aureus *to β-lactam antibiotics or antibiotics of other groups results in the generation of PMN-stimulating activity and whether this activity can be attributed to LTA.

**Methods:**

*S. aureus *were exposed to flucloxacillin, a β-lactam antibiotic or to the protein synthesis-inhibitors erythromycin and gentamicin, or to ciprofloxacin, a gyrase inhibitor. Supernatants of the antibiotic-treated bacteria were assayed for their LTA content and for their effect on PMN functions.

**Results:**

We observed that exposure of *S. aureus *to flucloxacillin and, to a lesser degree to ciprofloxacin, but not to erythromycin or gentamicin led to LTA release. Co-incubation of neutrophil granulocytes with LTA-containing supernatants led to PMN activation as assed by morphological changes, release of IL-8, delay of spontaneous apoptosis and enhanced phagocytic activity. Depletion of LTA from the supernatants markedly reduced their PMN-activating capacity.

**Conclusion:**

The findings suggest that, via the activation of PMN, antibiotic-induced LTA release from *S. aureus *leads to enhanced antimicrobial activity of the innate immune defense mechanisms.

## Background

Gram-positive bacteria are responsible for 50 % of bacterial infections. *Stapyhylococcus aureus *(*S. aureus*) is a major human pathogenic gram-positive bacterium causing a diverse array of diseases ranging from minor skin and wound diseases to more serious and life-threatening diseases like pneumonia, endocarditis and arthritis [1]. *S. aureus*, as all gram-positive bacteria, has a thick cell wall of peptidoglycan which covers a thin cytoplasmic membrane. Several compounds such as teichoic acid, lipoglycans, polysaccharides and lipoteichoic acid (LTA) are incorporated in the peptidoglycan exoskeleton. LTA is a major immunostimulatory component of *S. aureus*. In a previous study we demonstrated that highly purified LTA from *S. aureus *directly activated polymorphonuclear neutrophil granulocytes (PMN) resulting in morphological changes, shedding of CD62L, degranulation, cytokine release, priming of fMLP-mediated oxidative burst and delay in the spontaneous apoptosis [2]. Neutrophil granulocytes are phagocytes of the innate immune system that participate in inflammatory reactions as first line of defense against microbial pathogens. Since neutropenic patients are prone to *S. aureus *infections, and neutrophil granulocytes are able to phagocytose and kill *S. aureus in vitro *[3], PMN have long been thought to provide significant host defense against this pathogen.

Previous studies from other laboratories indicated that certain antibiotics mainly β-lactam compounds induce LTA release from the gram-positive bacteria *Streptococcus pneumoniae *[4] and *S. aureus *[5,6] whereas protein synthesis inhibitors did not lead to enhanced LTA release [6]. Based on the two previous findings that i) supernatants of β-lactam antibiotic-treated *S. aureus *contain LTA and ii) purified *S. aureus *LTA can activate neutrophils, in the present study we addressed the hypothesis whether supernatants of β-lactam antibiotic-treated *S. aureus *can directly activate neutrophil granulocytes. The experiments revealed a marked activation of PMN by the supernatants, i.e. the cells acquired an activated cell shape, released IL-8, their spontaneous apoptosis was delayed and their phagocytic activity was enhanced. The major neutrophil activating component in the supernatants was LTA since depletion of LTA markedly reduced the PMN-activating effect of the supernatants. These data suggest that treatment of *S. aureus *infections with β-lactam antibiotics, in addition to the direct antibacterial activity, has an activating effect on the innate immune system.

## Methods

### Isolation of human peripheral blood neutrophil granulocytes

Peripheral blood was collected by venipuncture from healthy adult volunteers using lithium-heparin. Blood was layered on a two-layer density gradient consisting of lymphocyte separation medium 1077 (upper layer, PAA, Pasching, Austria) and Histopaque^® ^1119 (bottom layer, Sigma, Deisenhofen, Germany) and centrifuged for 5 min at 300 × g followed by 20 min at 800 × g. Cells from the upper layer consisting mainly of lymphocytes and monocytes were discarded. The granulocyte-rich lower layer was collected leaving the erythrocyte pellet at the bottom of the tube. Granulocytes were washed once in PBS, resuspended in complete medium (RPMI 1640 medium, (Sigma) supplemented with 50 μM 2-mercaptoethanol, 2 mM L-glutamine, 10 mM HEPES (all from Biochrom, Berlin, Germany)) and 10 % fetal calf serum (FCS, Gibco, Karlsruhe, Germany) and further fractionated on a discontinuous Percoll^® ^(Amersham Biosciences, Uppsala, Sweden) gradient consisting of layers with densities of 1.105 g/ml (85 %), 1.100 g/ml (80 %), 1.087 g/ml (70 %), and 1.081 g/ml (65 %). After centrifugation for 20 min at 800 × g, the interface between the 80 and 70 % Percoll^® ^layers was collected, the cells were washed once in PBS and resuspended in complete medium to a concentration of 5 × 10^6^/per ml. All procedures were conducted under sterile conditions at room temperature. The cell preparations contained > 99.9 % granulocytes as determined by morphological examination of > 1,000 cells on Giemsa stained cytocentrifuge (Shandon, Pittsburgh, PA) slides. Cell viability was > 99 %, as determined by trypan blue exclusion.

### Cell culture

PMN were cultured at a concentration of 5 × 10^6 ^cells per ml in complete medium (see above) at 37°C in a humidified atmosphere containing 5 % CO_2 _in tissue culture plates with 96 flat-bottom wells (Greiner, Frickenhausen, Germany). The cells were coincubated for the indicated time points with the bacterial supernatants (see below) or with 10 μg/ml of highly purified *S. aureus *LTA [7]. The morphology of PMN in the cell culture was monitored using an invert microscope and documented by digital imaging (Carl Zeiss, Jena, Germany).

### *Staphylococcus aureus*

*S. aureus *DSM 20233 strain (DSMZ, Braunschweig, Germany) was cultured using Columbia sheep (COS) blood agar plate (Bio Mérieux, Marcy I'Etoite, France) and incubated at 37°C. The bacteria were resuspended in complete medium and quantified by densitometry (Densimat, Bio Mérieux) using McFarland standards (McFarland of 0.5 corresponds to approximately 1.5 × 10^8 ^bacteria/ml).

### Morphological assessment of PMN apoptosis

Apoptotic PMN exhibit typical morphological features such as condensation of the nuclear chromatin and separation of darkly stained pyknotic nuclear lobes [8,9]. Using these morphological criteria the percentage of apoptotic PMN was determined in cultures exposed to *S. aureus *supernatants. Nuclear morphology was assessed on Giemsa-stained cytocentrifuge slides. Cell morphology was examined under oil immersion light microscopy (Carl Zeiss). A minimum of 200 cells/slide were examined and graded as apoptotic/nonapoptotic.

### Determination of IL-8 in the supernatants of PMN cultures

Cell free supernatants from PMN cultures (5 × 10^6 ^cells/ml) were collected after indicated time points and stored at -20°C until cytokine determination. IL-8 was measured using an enzyme-linked immunosorbent assay (ELISA, CytoSets™, Biosource, Camarillo, CA) according to the manufacturer's instructions. The detection limit was 30 pg/ml.

### Analysis of the phagocytic activity of PMN

To determine the phagocytic activity, PMN were incubated for 75 min at 37°C in a 96-well flat-bottom tissue culture plate (i) in medium without antibiotics, or (ii) in medium with flucloxacillin (FLU, 2.5 μg/ml, corresponding to 20 × minimal inhibitory concentration (MIC), Stapylex^® ^Injection, GlaxoSmithKline, Munich, Germany), or (iii) in medium containing highly purified *S. aureus *LTA [7] (10 μg/ml) or (iv) in a supernatant of flucloxacillin-treated *S. aureus *(see below). Subsequently, viable *S. aureus *of three strains (the reference strain DSM 20233 and two clinical isolates) were added at a bacterium to PMN ratio of 3:1 and the uptake of bacteria was assessed after incubation for 30 min at 37°C. The number of PMN with phagocytosed *S. aureus *was assessed on Giemsa-stained cytocentrifuge slides under oil immersion light microscopy. The samples were blinded and scored by two independent investigators. A minimum of 500 cells/slide were examined and the ratio of PMN with phagocytosed bacteria was determined.

### Generation of bacterial supernatants

The susceptibility of *S. aureus *to the four antibiotics flucloxacillin, erythromycin (ERY, Sigma), gentamicin (GEN, Sigma) and ciprofloxacin (CIP, Ciprobay^® ^100, Bayer Vital, Leverkusen, Germany) was determined by assessing the minimal inhibitory concentrations (MIC)s. In case of GEN the E-test^® ^(Viva Diagnostics, Cologne, Germany) was used. The MIC of the FLU, ERY and CIP was determined using a two-fold dilution starting with 2 μg/ml [10]. The MIC for GEN was 0.75 μg/ml, for CIP 0.25 μg/ml, for FLU 0.125 μg/ml and for ERY 1 μg/ml.

Bacterial supernatants were generated as described [5]. *S. aureus *concentration was determined by the McFarland values. A McFarland of 0.5 corresponds to approximately 1.5 × 10^8 ^bacteria/ml. *S. aureus *was incubated in complete medium at a starting concentration of 1 × 10^7^/ml for two hours at 37°C. Subsequently, antibiotics were added at a concentration of 1 × MIC or 20 × MIC for 4 hours. After incubation, the supernatants were collected and sterile filtered (0.45 μm, Millipore, Schwalbach, Germany).

To check the killing effect of the antibiotics, bacterial suspensions were spread on COS blood agar plates after the 4 hours incubation with antibiotics. Colonies were counted after an overnight incubation (Table 1). Flucloxacillin, gentamicin and ciprofloxacin exerted a marked bactericidal effect whereas erythromycin was basically bacteriostatic.

**Table 1:** Effect of various antibiotics on the bacterial counts in cultures of *S. aureus*. *S. aureus *(DSM 20233 strain) bacteria were incubated in complete medium for 4 hours in the presence of antibiotics. Subsequently, bacterial suspensions were spread on COS blood agar plates. Colonies were counted after an overnight incubation.

Treatment	bacteria/ml
starting concentration	4 × 10^6^
4 hours without antibiotics	5.1 × 10^7^
4 hours with 1 × MIC flucloxacillin	1.8 × 10^5^
4 hours with 1 × MIC ciprofloxacin	2.5 × 10^3^
4 hours with 1 × MIC gentamicin	5.2 × 10^5^
4 hours with 1 × MIC erythromycin	8.3 × 10^5^
4 hours with 20 × MIC flucloxacillin	10
4 hours with 20 × MIC ciprofloxacin	0
4 hours with 20 × MIC gentamicin	0
4 hours with 20 × MIC erythromycin	5.9 × 10^5^

### Determination of LTA-content of bacterial supernatants

An ELISA was performed to determine the concentration of LTA in the bacterial supernatants [6]. Highly purified LTA of *S. aureus *at concentrations of 31 to 2000 ng/ml in complete medium was used to set a standard curve. The samples were incubated overnight at room temperature in a 96-well PolySorb™ immunoplate (Nunc, Wiesbaden, Germany). The plates were blocked with PBS + 0.5 % BSA + 0.05 % Tween 20 for one hour followed by three wash steps with PBS + 0.05 % Tween 20. For detection, a mouse IgG3 anti-LTA mAb (IgG3, clone 55, Hbt, AA Uden, The Netherlands) was added at a concentration of 1.2 μg/ml diluted in PBS + 0.5 % BSA. After an incubation for one hour at 37°C the plates were washed and incubated with 2 μg/ml goat-anti-mouse Ig-HRP conjugate (Dako, Hamburg, Germany) for 90 min. After three washing steps TMB substrate (BD Biosciences, Heidelberg, Germany) was added. The reaction was stopped after 15 min with 2 N H_2_SO_4 _(Merck, Darmstadt, Germany). The absorption was measured at 450 nm. The detection limit of the assay was 62 pg/ml.

### Depletion of LTA from bacterial supernatants

In order to investigate whether LTA was the PMN-activating component of the bacterial supernatants LTA was depleted from the supernatants. Protein G Sepharose 4 fast flow beads (Amersham Bioscience, Heidelberg, Germany) were coated with the anti-LTA mAb. 80 μl of a suspension of Protein G Sepharose beads were centrifuged at 15,300 × g for 20 sec, the pellet was washed three times with PBS and resuspended in 80 μl PBS. 40 μl of the Protein G Sepharose suspension were incubated with 70 μl anti-LTA mAb (antibody stock concentration > 200 μg/ml) for one hour at 4°C. The remaining 40 μl of Protein G Sepharose suspension were incubated with PBS as control in a glass vial for one hour at 4°C under continuous shaking. The suspensions were centrifuged and the pellets (Protein G-Ab-complex) were washed four times with PBS. To deplete LTA from the supernatants, pellets were resuspended in 250 μl supernatants from flucloxacillin-treated (20 × MIC) bacteria and incubated for 45 min at 4°C under continuous shaking. The Sepharose bead suspensions were centrifuged and the supernatants were taken. The depletion procedure was repeated once more with freshly prepared anti-LTA-Sepharose beads. Finally, the LTA depleted supernatant was centrifuged two times to remove the Sepharose beads. The efficacy of the LTA depletion was 80 % as assessed in a LTA-ELISA by measuring the LTA-content in the supernatants before and after depletion. PMN were incubated with the LTA-depleted, control (only protein G Sepharose treated) or untreated supernatant.

### Statistical analysis

Data are presented as mean ± standard deviation (SD) and analyzed using JMP™ statistical software (Version 5.1 SAS Institute, Cary, NC) or GraphPadPrism^® ^(Version 4.01, San Diego, CA). Data were tested for homoscedasticity (homogenous variances of the data sets) with the Bartlett test and for normal distribution with the Shapiro-Wilks W-test. When both assumptions were met the data were analyzed by one-way analysis (ANOVA) and a post test, Tukey-Kramer for comparison of all pairs or Dunnett for pair wise comparison with the control. If the variances were not equal the transformation (log_10 _(x + 0.5)) was performed. When after the transformation the assumptions were fulfilled the data were analyzed by ANOVA as described before. However, if the assumptions were not met, a non-parametric analysis was performed, the Kruskal-Wallis for multiple comparisons test followed by a Dunns post test. Overall, differences were considered statistically significant at p < 0.05 and are indicated with an asterisk.

## Results

In this study the question was addressed whether exposure of *S. aureus *to β-lactam antibiotics or antibiotics of other groups leads to the generation of PMN-stimulating activity and whether this activity can be attributed to LTA. *S. aureus *bacteria were exposed to the β-lactam antibiotic flucloxacillin, to erythromycin or gentamicin, both protein synthesis-inhibitors, or to ciprofloxacin, a gyrase inhibitor. In preliminary experiments it was tested whether the antibiotics alone exhibited an effect on PMN. Flucloxacillin, gentamicin, erythromycin and ciprofloxacin had no effect on PMN regarding their morphology, apoptosis and cytokine release (data not shown).

### Neutrophil granulocytes acquire an activated cell morphology upon exposure to supernatants of antibiotic-treated *S. aureus*

**Figure 1:**
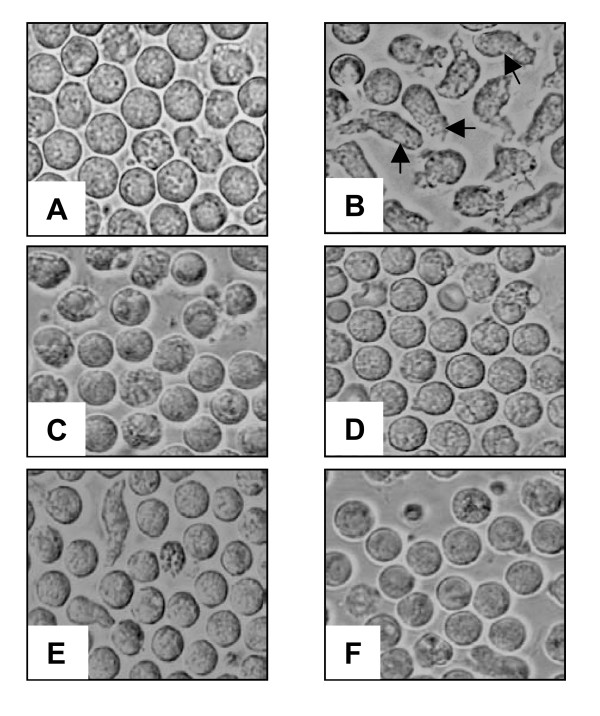
**PMN acquire an activated cell shape after exposure to bacterial supernatants**. PMN (5 × 10^6^/ml) were incubated overnight in medium alone (A) or in supernatants of *S. aureus *exposed to flucloxacillin 20 × MIC (B), erythromycin 20 × MIC (C), gentamicin 20 × MIC (D), ciprofloxacin 1 × MIC (E) or ciprofloxacin 20 × MIC (F). Elongated cell morphology (arrows) is a sign for activation of PMN (original magnification 200 ×).

Neutrophil activation is associated with marked changes in cell morphology. Typically, activated PMN appear as elongated, motile cells [11,12]. PMN were incubated with supernatants of antibiotic-exposed *S. aureus*. We observed that PMN acquired such activated elongated cell shape upon exposure to supernatants of flucloxacillin-treated *S. aureus *(Fig. 1B). A moderate activation was also observed when PMN were coincubated with supernatants of ciprofloxacin-exposed (1 × MIC) bacteria (Fig. 1E). Interestingly, PMN incubated with supernatants of 20 × MIC ciprofloxacin-exposed bacteria did no show activated cell morphology (Fig. 1F). No activated cells were seen in cultures of PMN after co-incubation with supernatants of erythromycin- or gentamicin-exposed bacteria (Fig. 1C, D). These morphological observations indicate that PMN-activating substance(s) are released from *S. aureus *upon treatment with flucloxacillin and, to a lesser degree, with ciprofloxacin.

### Neutrophil granulocytes release IL-8 after co-incubation with supernatants of antibiotic-exposed *S. aureus*

**Figure 2:**
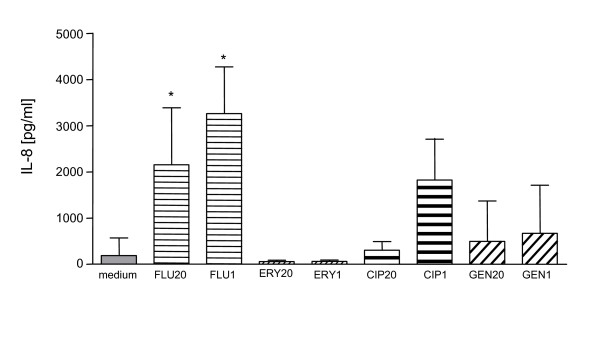
**Bacterial supernatants induce the secretion of IL-8 by PMN**. Neutrophils were incubated at 5 × 10^6^/ml overnight in medium alone or with supernatants of antibiotic-exposed bacteria. The IL-8 content was measured by ELISA. The results are mean ± SD of three to eight experiments. Significant differences between treated and non-treated cultures are indicated (Kruskal-Wallis test, Dunns test). FLU20 = flucloxacillin 20 × MIC; FLU1 = flucloxacillin 1 × MIC; ERY20 = erythromycin 20 × MIC; ERY1 = erythromycin 1 × MIC; CIP20 = ciprofloxacin 20 × MIC; CIP1 = ciprofloxacin 1 × MIC; GEN20 = gentamicin 20 × MIC; GEN1 = gentamicin 1 × MIC.

Neutrophil granulocytes are the major source of the proinflammatory chemokine IL-8 [13]. Therefore, IL-8 release by PMN was assessed upon exposure to supernatants of antibiotic-treated *S. aureus *cultures overnight. Supernatants of flucloxacillin-exposed bacteria induced high levels of IL-8 secretion (Fig. 2). A lower level of IL-8 secretion was induced by supernatants of ciprofloxacin-exposed *S. aureus*. Supernatants of gentamicin- or erythromycin-treated *S. aureus *did not induce IL-8 release in cultures of human PMN (Fig. 2). These data indicate that IL-8 release inducing substance(s) are in the supernatants of flucloxacillin- and ciprofloxacin-exposed bacteria.

### PMN apoptosis is delayed in the presence of supernatants of antibiotic-exposed *S. aureus*

**Figure 3:**
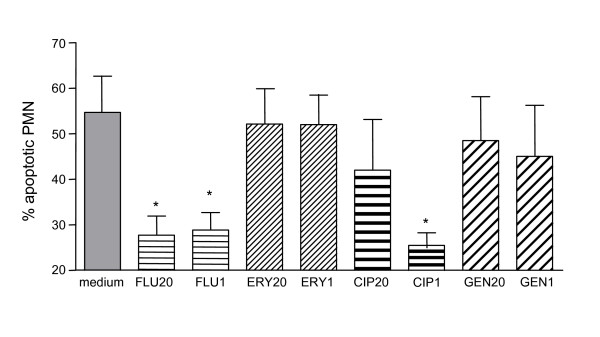
**Bacterial supernatants delay the apoptosis of neutrophil granulocytes**. PMN (5 × 10^6^/ml) were cultured in medium or with supernatants of antibiotic-exposed bacteria. After overnight co-incubation the percentage of apoptotic PMN was determined by microscopical evaluation of > 200 cells on cytocentrifuge preparations stained with Giemsa. Results are mean ± SD of three to nine experiments. Significant differences between treated and non-treated cultures are indicated (Kruskal-Wallis test, nns test). FLU20 = flucloxacillin 20 × MIC; FLU1 = flucloxacillin 1 × MIC; ERY20 = erythromycin 20 × MIC; ERY1 = erythromycin 1 × MIC; CIP20 = ciprofloxacin 20 × MIC; CIPi1 = ciprofloxacin 1 × MIC; GEN20 = gentamicin 20 × MIC; GEN1 = gentamicin 1 × MIC.

Neutrophils undergo constitutive apoptosis when aged both *in vivo *and *in vitro*. Apoptotic PMN exhibit typical morphological features such as cell shrinkage and condensation of the nucleus [8,9]. Using these morphological criteria the percentage of apoptotic PMN was determined in cultures exposed to *S. aureus *supernatants. The percentage of apoptotic cells was strongly reduced when neutrophils were coincubated with supernatants of flucloxacillin- (1 × or 20 × MIC) or ciprofloxacin- (1 × MIC) exposed bacteria as compared to the apoptosis rate of PMN cultured in medium alone (Fig. 3). Supernatants of gentamicin- or erythromycin-exposed *S. aureus *did not affect the apoptosis of PMN (Fig. 3). It is known that constitutive apoptosis is a feature of resting/non-activated PMN. Once PMN are activated and engaged in function, their apoptosis is delayed. Therefore, the inhibition of PMN apoptosis through supernatants of flucloxacillin- and ciprofloxacin-exposed *S. aureus *is a further proof for PMN-activating substance(s) in these supernatants.

### LTA is present in the PMN-activating bacterial supernatants

**Figure 4:**
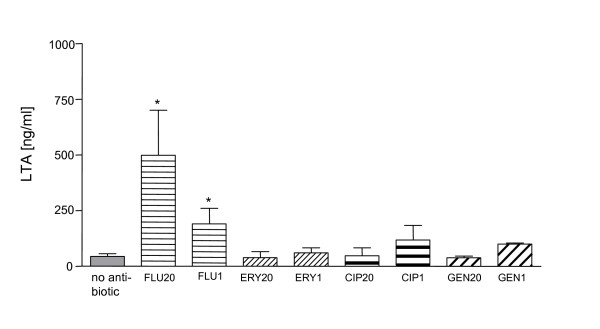
***S. aureus *release LTA after treatment with antibiotics**. *S. aureus *were exposed to antibiotics for four hours. The bacterial supernatants were collected and sterile filtered. The LTA content of the supernatants was measured with an LTA-specific ELISA. Data are expressed as mean ± SD of three experiments. Significant differences between treated and non-treated cultures are indicated (transformation log_10_(x + 0.5), ANOVA, Dunnetts post test). FLU20 = flucloxacillin 20 × MIC; FLU1 = flucloxacillin 1 × MIC; ERY20 = erythromycin 20 × MIC; ERY1 = erythromycin 1 × MIC; CIP20 = ciprofloxacin 20 × MIC; CIP1 = ciprofloxacin 1 × MIC; GEN20 = gentamicin 20 × MIC; GEN1 = gentamicin 1 × MIC.

Data presented above indicate that supernatants of flucloxacillin-exposed and, to a lesser degree, of ciprofloxacin-exposed *S. aureus *activate neutrophil granulocytes. We addressed the question which component of these supernatants is responsible for the activation of granulocytes. A likely candidate was LTA, since in a previous study purified LTA was observed to activate PMN [2]. The LTA content was measured in supernatants of *S. aureus *with or without antibiotic exposure. High LTA content was detected in supernatants of flucloxacillin-exposed bacteria (Fig. 4). A moderate level of LTA was also present in ciprofloxacin-exposed (1 × MIC) *S. aureus *supernatants. The other antibiotics did not significantly induce LTA release from *S. aureus*. Since the LTA content of the supernatants positively correlates with the PMN-activating capacity, the data strongly suggest that LTA is at least one of the PMN-activating components in the supernatants.

### Depletion of LTA from the supernatant reduces PMN activation

**Figure 5:**
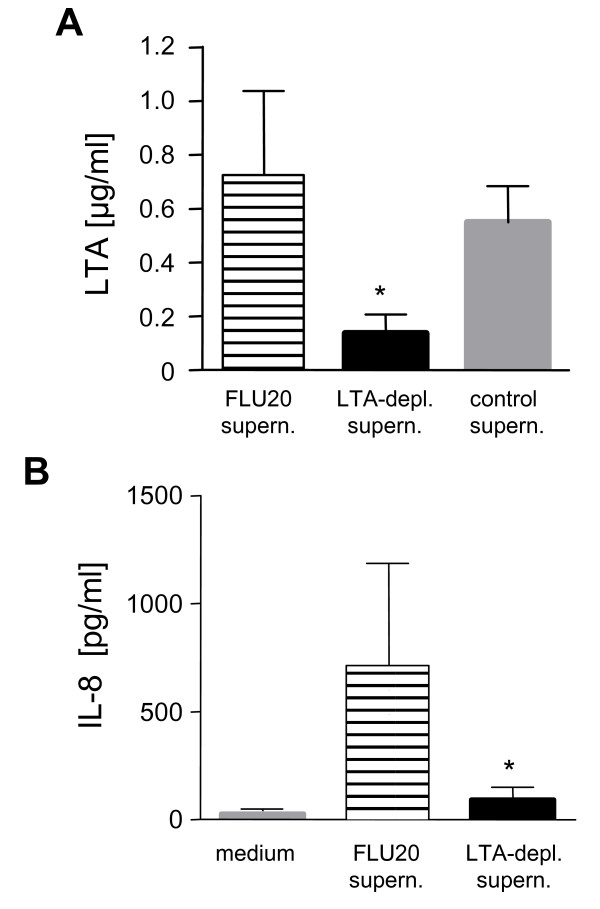
**Depletion of LTA results in the reduction of PMN-activating capacity of *S. aureus *supernatants**. A) LTA was depleted from flucloxacillin-exposed (20 × MIC, FLU20) *S. aureus *supernatants as described in Materials and Methods. LTA content was measured before and after LTA-depletion using an ELISA. Data are expressed as mean ± SD of three experiments. *Asterisk indicates significant difference (transformation log_10_(x + 0.5), ANOVA, Dunnetts post test). B) Neutrophils were incubated at 5 × 10^6^/ml overnight in medium alone, with the LTA-containing or the LTA-depleted bacterial supernatants. The IL-8 content in the PMN supernatants was measured using ELISA. Data are expressed as mean ± SD of three experiments. Significant difference was analyzed after a transformation log_10_(x + 0.5) by ANOVA and Tukey-Kramer post test.

Depletion experiments were carried out to investigate whether LTA is the PMN-activating component of *S. aureus *supernatants. LTA was depleted from the supernatant with the highest LTA content (flucloxacillin 20 × MIC) as described in the Methods. Using this technique the LTA content of the supernatant was reduced by 80 % (Fig. 5A). Co-incubation of PMN with LTA-depleted supernatants led to a marked decrease in IL-8 release as compared to incubation with LTA-containing supernatants (Fig. 5B). The number of cells with activated morphology was also reduced when the cells were incubated with the LTA-depleted supernatants (data not shown). These data indicate that LTA is a major PMN-activating component in supernatants of flucloxacillin-exposed *S. aureus*.

### LTA-containing bacterial supernatants enhance the phagocytic activity of neutrophil granulocytes

**Figure 6:**
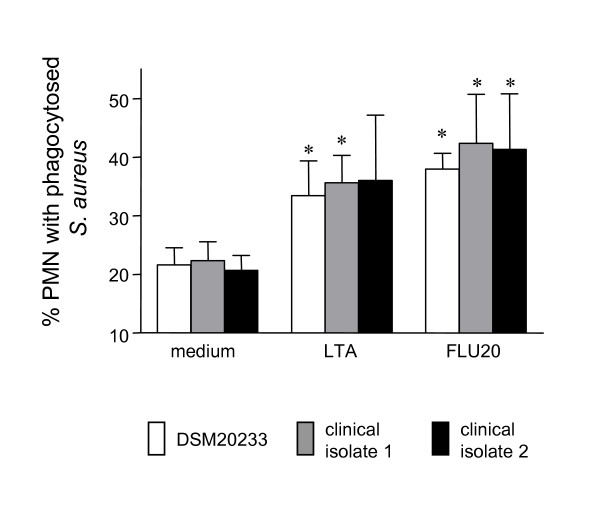
**LTA enhances the phagocytic activity of PMN**. Neutrophil granulocytes were incubated for 75 minutes at 37°C in medium alone or in the presence of LTA (10 μg/ml) or in the presence of a supernatant of flucloxacillin (20 × MIC)-exposed *S. aureus*. Subsequently, viable *S. aureus *(3:1; bacteria:PMN) of three strains (the reference strain DSM 20233 and two clinical isolates) were added and the cells incubated for an additional 30 minutes at 37°C. The number of PMN with phagocytosed *S. aureus *was assessed on Giemsa-stained cytocentrifuge slides under oil immersion light microscopy. Data (mean ± SD) of three independent experiments are shown. Significant differences (ANOVA, Dunnets test) to unstimulated PMN are indicated (*)

Phagocytosis of pathogenic microorganisms is one of the most important effector functions of neutrophil granulocytes. Having observed that LTA-containing supernatants of flucloxacillin-treated bacteria activate PMN, we addressed the question whether these activated granulocytes are more potent regarding the phagocytosis of *S. aureus*. Neutrophil granulocytes were incubated in medium alone, stimulated with LTA or LTA-containing supernatants of flucloxacillin- (20 × MIC) exposed bacteria for 75 min at 37°C. Subsequently, viable *S. aureus *of three strains (the reference strain DSM 20233 and two clinical isolates) were added at a bacterium to PMN ratio of 3:1 and the uptake of bacteria was assessed in a 30 min phagocytosis assay. Both highly purified LTA and supernatants of flucloxacillin-exposed bacteria enhanced markedly the phagocytic activity of PMN (Fig. 6). These data indicate that the LTA-containing bacterial supernatants not only activate PMN as assessed by morphological changes, IL-8 release and apoptosis delay but also enhance their phagocytic activity.

## Discussion

We report that *S. aureus *release neutrophil-activating substance(s) upon exposure to the β-lactam antibiotic flucloxacillin and, to a lesser degree, to the gyrase inhibitor ciprofloxacin. We show data that supernatants of antibiotic-exposed *S. aureus *induce morphological changes and IL-8 release in cultures of highly purified human PMN. Moreover, exposure to *S. aureus *supernatants delays the spontaneous apoptosis and enhances the phagocytic capacity of PMN. We present evidence that lipoteichoic acid (LTA) is a major PMN-activating component present in supernatants of antibiotic-exposed *S. aureus*.

PMN are phagocytic cells that participate in innate immune responses as a first line of defense against microbial pathogens. *S. aureus*, a gram-positive bacterium, is known to activate PMN functions leading to phagocytosis and in most cases to killing of the ingested bacteria. However, it is still poorly characterized which component of *S. aureus *is the most potent PMN-activating molecule. Compared to LPS from gram-negative bacteria [14], the molecular basis of LTA-mediated PMN activation is poorly understood. Although in a previous study LTA was not identified as PMN-activating component of *S. aureus *supernatants [15] we have recently reported, that highly purified *S. aureus *LTA exerts a direct stimulatory effect on human PMN in a TLR2- and CD14-dependent manner [2]. LTA is a component of the cell-membrane of gram-positive bacteria. Treatment with β-lactam antibiotics that interfere with the cell wall biosynthesis of the bacteria was reported to induce release of LTA from *S. aureus *[5]. Therefore, we hypothesized, that LTA-containing supernatants of β-lactam-antibiotic-exposed *S. aureus *exert a stimulatory effect on human PMN. The data presented in this work proved the hypothesis. Upon exposure to supernatants of flucloxacillin-treated *S. aureus *PMN acquired an activated cell shape and released significant amounts of IL-8. After pathogen challenge, IL-8 is one of the most important chemotactic factors that mediate local neutrophil recruitment [13]. The autocrine production of IL-8 by activated PMN is regarded as an amplifying loop to attract more neutrophils to the site of infection [16]. In an *in vivo *study intranasal application of LTA led to the recruitment of PMN in the murine lung [17]. Although it can not be excluded that LTA exerts a direct chemotactic activity on PMN, the massive neutrophil accumulation in the murine model is likely to be a result of chemotactic factors released locally upon exposure to LTA. Indeed, an increased level of KC, the murine functional homologue of IL-8, was measured in the lung after treatment with LTA [17]. Our previous data showed a high IL-8 release by PMN after exposure to LTA [2]. This finding indicates that after exposure to LTA and to LTA-containing supernatants, PMN-derived IL-8 participates in the recruitment of more PMN to the site of infection.

*S. aureus *and LTA were previously shown to stimulate the cytokine production of monocytes [18]. These cytokines include several proinflammatory cytokines which all are known to possess the capacity to stimulate PMN. The goal of our study was to investigate the direct effects of antibiotic-exposed *S. aureus *supernatants on PMN. Therefore, indirect effects of LTA mediated by monocytes had to be ruled out, i.e. special care had to be taken to avoid monocyte contamination. Our technique for PMN isolation resulted in a highly pure granulocyte population containing solely two or less monocytes per 10,000 granulocytes [2]. The marked biological effect of LTA-containing supernatants on this highly pure PMN population strongly argues against a possible indirect effect mediated by soluble factors released by contaminating leukocytes other then PMN.

PMN are inherently short-lived cells with a half-life of only about 6–10 hours, after which they undergo spontaneous apoptosis [8]. The life span of neutrophils can be extended *in vitro *by incubation with either proinflammatory cytokines including GM-CSF and G-CSF, IL-8, IL-1β, or bacterial products such as LPS and fMLP [19,20]. This implies that PMN die rapidly via apoptosis if not engaged in function. After activation, however, inflammatory PMN have an extended life span that enables these cells to execute their antimicrobial effector functions. Co-incubation of PMN with purified *S. aureus *LTA was shown to activate PMN leading to enhanced life span [2]. Here we show that upon treatment with flucloxacillin or ciprofloxacin but not with gentamicin or erythromycin *S. aureus *release substances into the supernatant that delay PMN apoptosis. Since the apoptosis-delaying activity of the supernatants correlated with their LTA-content we conclude that antibiotics that induce LTA release can expand the functional longevity of neutrophil granulocytes.

The results presented here are in line with the data of van Langevelde et al. [6] concerning the different effects of antibiotics on the LTA release by *S. aureus*. Flucloxacillin exposure induced LTA release by *S. aureus *whereas erythromycin and gentamicin did not. The exact mechanism of how antibiotic treatment leads to LTA release is still unknown. Pollack et al. suggested that membrane vesicles containing LTA are formed and extruded by the action of β-lactam antibiotics. This way of LTA-release appears to be independent of bacteriolysis [21]. On the other hand β-lactam antibiotics inhibit the bacterial cell wall synthesis. The accumulation of cell wall precursors was reported to reduce the stability of LTA in the cell membrane [22]. As LTA binds and inhibits autolytic enzymes in the cell wall [23], an enhanced release of LTA could reduce the inhibition of autolytic enzymes and, consequently, could result in bacteriolysis. We showed that treatment with a low concentration of ciprofloxacin also exhibited a moderate increase in LTA release by *S. aureus*. As described previously with gram-negative bacteria, ciprofloxacin treatment can induce endotoxin release [24]. Others showed that at low concentrations trovafloxacin, another quinolone, induced the release of LTA and teichoic acids from the gram-positive bacterium *Streptococcus pneumoniae *[4]. Although the primary mechanism of quinolone action is the inhibition of DNA gyrase activity, it was reported that exposure to low concentrations of quinolones led to the induction of bacteriolysis [25]. This mechanism is a likely explanation why low-dose ciprofloxacin treatment of *S. aureus *resulted in an enhanced release of LTA and, consequently, to activation of PMN.

In the supernatants of antibiotic-exposed *S. aureus *the LTA concentration was below 1 μg/ml. Still, regarding their PMN-activating capacity, these supernatants were as potent as 10 μg/ml purified LTA. On the one hand, a reduction of biological activity during the purification process of LTA may be a reason for this discrepancy. Indeed, various biochemical alterations which could be happen during purification [7] or through enzymatic degradation [26] were shown to reduce the cytokine-inducing capacities of cell wall components. On the other hand, LTA is certainly one of several bacterial constituents released after antibiotic-treatment from *S. aureus*. Cell wall components such as peptidoglycan and teichoic acid were shown to be present in bacterial supernatants [27]. Moreover, bacterial supernatants likely contain several additional bacterial products. These various factors often act on leukocytes in a synergistic way, as demonstrated for muramyldipeptide (*N*-acetylmuramyl-L-alanyl-D-isoglutamine) with LTA [28]. Muramyldipeptide is the minimal essential structure of peptidoglycan exerting biological activity. Therefore, the high biological activity of supernatants from antibiotic-exposed *S. aureus *is likely a consequence of synergistic actions of various bacterial components. However, our depletion experiments clearly indicated that LTA is a major and essential PMN-activating component of *S. aureus *supernatants.

Once recruited at inflamed sites, PMN recognize and phagocytose gram-positive bacteria resulting in the activation of these antimicrobial effector cells [3]. Here it was shown that, in addition to highly purified LTA, LTA-containing supernatants of *S. aureus *also upregulated the phagocytic activity of PMN. Therefore, LTA enhances the ability of PMN to kill the bacteria and to clear the infection. Treatment with β-lactam antibiotics and, to a lesser degree with gyrase inhibitors, can enhance the efficiency of innate immune mechanisms through the activation of neutrophil granulocytes. In addition, the prolonged life span permits PMN to exert their enhanced ability to fight against gram-positive bacteria for a longer period of time.

Activated granulocytes are essential components of the antimicrobial innate defense. Nevertheless, it should be kept in mind that granulocytes can be the cause of severe pathological conditions through tissue damage [29]. Since local LTA release can lead to PMN recruitment [17,30], local PMN-mediated reactions could trigger severe pathology if the bacterial infection is not efficiently cleared. However, our data show that antibiotic-treated rather than intact/viable *S. aureus *release significant amounts of LTA. The antibiotics that induce LTA-release are effective to kill the bacteria and, therefore, limit the persistence of these pathogens in the infected tissue. Therefore, the beneficial effects of antibiotic-induced LTA-release and the consequent PMN-activation are likely to exceed the potential tissue destruction through activated granulocytes.

## Conclusion

The presented data indicate that LTA is one major PMN-activating component in the supernatants of antibiotic-treated *S. aureus*. Treatment of *S. aureus *infections with β-lactam antibiotics that induce LTA release has, in addition to the direct antibacterial activity, an activating effect on the innate immune system. Local LTA release can lead to recruitment and activation of neutrophil granulocytes at the site of infection. The extended life span and enhanced phagocytic activity of PMN can contribute to a more efficient resolution of the infection.

## Abbreviations

CIP, ciprofloxacin; ERY, erythromycin; FLU, flucloxacillin; GEN, gentamicin; LTA, lipoteichoic acid; MIC, minimal inhibitory concentration; PMN, polymorphonuclear neutrophil granulocytes

## Declaration of competing interests

The author(s) declare that they have no competing interests.

## Authors' contributions

SL carried out most experiments with human granulocytes, measured and depleted LTA in culture supernatants and drafted the manuscript. AS cultivated the *S. aureus *strains. CZ analyzed PMN apoptosis. SM and TH purified LTA from *S. aureus *and contributed to the experimental design. WS critically revised the manuscript for important intellectual content. TL conceived of the study, participated in its design and coordination and helped to draft the manuscript

## References

[B1] Lowy FD (1998). *Staphylococcus aureus *infections. N Engl J Med.

[B2] Lotz S, Aga E, Wilde I, van Zandbergen G, Hartung T, Solbach W, Laskay T (2004). Highly purified lipoteichoic acid activates neutrophil granulocytes and delays their spontaneous apoptosis via CD14 and TLR2. J Leukoc Biol.

[B3] Vandenbroucke-Grauls CM, Thijssen HM, Verhoef J (1984). Interaction between human polymorphonuclear leucocytes and *Staphylococcus aureus *in the presence and absence of opsonins. Immunology.

[B4] Stuertz K, Schmidt H, Eiffert H, Schwartz P, Mader M, Nau R (1998). Differential release of lipoteichoic and teichoic acids from *Streptococcus pneumoniae *as a result of exposure to beta-lactam antibiotics, rifamycins, trovafloxacin, and quinupristin-dalfopristin. Antimicrob Agents Chemother.

[B5] Utsui Y, Ohya S, Takenouchi Y, Tajima M, Sugawara S, Deguchi K, Suginaka H (1983). Release of lipoteichoic acid from *Staphylococcus aureus *by treatment with cefmetazole and other beta-lactam antibiotics. J Antibiot (Tokyo).

[B6] van Langevelde P, van Dissel JT, Ravensbergen E, Appelmelk BJ, Schrijver IA, Groeneveld PH (1998). Antibiotic-induced release of lipoteichoic acid and peptidoglycan from *Staphylococcus aureus*: quantitative measurements and biological reactivities. Antimicrob Agents Chemother.

[B7] Morath S, Geyer A, Hartung T (2001). Structure-function relationship of cytokine induction by lipoteichoic acid from *Staphylococcus aureus*. J Exp Med.

[B8] Payne CM, Glasser L, Tischler ME, Wyckoff D, Cromey D, Fiederlein R, Bohnert O (1994). Programmed cell death of the normal human neutrophil: an in vitro model of senescence. Microsc Res Tech.

[B9] Squier MK, Sehnert AJ, Cohen JJ (1995). Apoptosis in leukocytes. J Leukoc Biol.

[B10] Burkhardt F (1992). Mikrobiologische Diagnostik.

[B11] Klut ME, Whalen BA, Hogg JC (1997). Activation-associated changes in blood and bone marrow neutrophils. J Leukoc Biol.

[B12] Stickle JE (1996). The neutrophil. Function, disorders, and testing. Vet Clin North Am Small Anim Pract.

[B13] Cassatella MA (1999). Neutrophil-derived proteins: selling cytokines by the pound. Adv Immunol.

[B14] Wilson ME (1985). Effects of bacterial endotoxins on neutrophil function. Rev Infect Dis.

[B15] Veldkamp KE, van Kessel KP, Verhoef J, van Strijp JA (1997). Staphylococcal culture supernates stimulate human phagocytes. Inflammation.

[B16] Gainet J, Chollet-Martin S, Brion M, Hakim J, Gougerot-Pocidalo MA, Elbim C (1998). Interleukin-8 production by polymorphonuclear neutrophils in patients with rapidly progressive periodontitis: an amplifying loop of polymorphonuclear neutrophil activation. Lab Invest.

[B17] von Aulock S, Morath S, Hareng L, Knapp S, van Kessel KP, van Strijp JA, Hartung T (2003). Lipoteichoic acid from *Staphylococcus aureus *is a potent stimulus for neutrophil recruitment. Immunobiol.

[B18] Ellingsen E, Morath S, Flo T, Schromm A, Hartung T, Thiemermann C, Espevik T, Golenbock D, Foster D, Solberg R, Aasen A, Wang J (2002). Induction of cytokine production in human T cells and monocytes by highly purified lipoteichoic acid: involvement of Toll-like receptors and CD14. Med Sci Monit.

[B19] Colotta F, Re F, Polentarutti N, Sozzani S, Mantovani A (1992). Modulation ofgranulocyte survival and programmed cell death by cytokines and bacterial products. Blood.

[B20] Lee A, Whyte MK, Haslett C (1993). Inhibition of apoptosis and prolongation of neutrophil functional longevity by inflammatory mediators. J Leukoc Biol.

[B21] Pollack JH, Ntamere AS, Neuhaus FC (1992). D-alanyl-lipoteichoic acid in *Lactobacillus casei*: secretion of vesicles in response to benzylpenicillin. J Gen Microbiol.

[B22] Tomasz A, Waks S (1975). Mechanism of action of penicillin: triggering of the pneumococcal autolytic enzyme by inhibitors of cell wall synthesis. Proc Natl Acad Sci USA.

[B23] Suginaka H, Shimatani M, Ogawa M, Kotani S (1979). Prevention of penicillin-induced lysis of *Staphylococcus aureus *by cellular lipoteichoic acid. J Antibiot (Tokyo).

[B24] Crosby HA, Bion JF, Penn CW, Elliott TS (1994). Antibiotic-induced release of endotoxin from bacteria in vitro. J Med Microbiol.

[B25] Vincent S, Glauner B, Gutmann L (1991). Lytic effect of two fluoroquinolones, ofloxacin and pefloxacin, on *Escherichia coli *W7 and its consequences on peptidoglycan composition. Antimicrob Agents Chemother.

[B26] Timmerman CP, Mattsson E, Martinez-Martinez L, De GL, van Strijp JA, Verbrugh HA, Verhoef J, Fleer A (1993). Induction of release of tumor necrosis factor from human monocytes by staphylococci and staphylococcal peptidoglycans. Infect Immun.

[B27] Tuomanen E, Liu H, Hengstler B, Zak O, Tomasz A (1985). The induction of meningeal inflammation by components of the pneumococcal cell wall. J Infect Dis.

[B28] Yang S, Tamai R, Akashi S, Takeuchi O, Akira S, Sugawara S, Takada H (2001). Synergistic effect of muramyldipeptide with lipopolysaccharide or lipoteichoic acid to induce inflammatory cytokines in human monocytic cells in culture. Infect Immun.

[B29] Witko-Sarsat V, Rieu P, Scamps-Latscha B, Lesavre P, Halbwachs-Mecarelli L (2000). Neutrophils: molecules, functions and pathophysiological aspects. Lab Invest.

[B30] Leemans JC, Heikens M, van Kessel KP, Florquin S, van der Poll T (2003). Lipoteichoic acid and peptidoglycan from *Staphylococcus aureus *synergistically induce neutrophil influx into the lungs of mice. Clin Diagn Lab Immunol.

